# Role of non-coding RNA networks in leukemia progression, metastasis and drug resistance

**DOI:** 10.1186/s12943-020-01175-9

**Published:** 2020-03-12

**Authors:** Ajaz A. Bhat, Salma N. Younes, Syed Shadab Raza, Lubna Zarif, Sabah Nisar, Ikhlak Ahmed, Rashid Mir, Sachin Kumar, Surender K. Sharawat, Sheema Hashem, Imadeldin Elfaki, Michal Kulinski, Shilpa Kuttikrishnan, Kirti S. Prabhu, Abdul Q. Khan, Santosh K. Yadav, Wael El-Rifai, Mohammad A. Zargar, Hatem Zayed, Mohammad Haris, Shahab Uddin

**Affiliations:** 1 Translational Medicine, Sidra Medicine, P.O. Box 26999, Doha, Qatar; 2 Department of Biomedical Science, College of Health Sciences, Qatar University, Doha, Qatar; 3 Translational Research Institute, Academic Health System, Hamad Medical Corporation, P.O. Box 3050, Doha, Qatar; 4 Laboratory for Stem Cell & Restorative Neurology, Era’s Lucknow Medical College and Hospital, Lucknow, Uttar Pradesh India; 5 Department of Medical Lab Technology, Faculty of Applied Medical Sciences, University of Tabuk, Tabuk, Saudi Arabia; 6 Department of Medical Oncology, Dr. B. R. Ambedkar Institute Rotary Cancer Hospital, All India Institute of Medical Sciences, New Delhi, India; 7 Department of Biochemistry, Faculty of Science, University of Tabuk, Tabuk, Saudi Arabia; 8 Department of Surgery, University of Miami, Miami, Florida USA; 9 Department of Biotechnology, Central University of Kashmir, Ganderbal, Jammu and Kashmir India; 10 Laboratory Animal Research Center, Qatar University, Doha, Qatar

**Keywords:** Cancer, Circular RNAs, Chromatin, Drug resistance, Epigenetics, Gene regulation, Long non-coding RNAs, MicroRNAs, Metastasis, Signaling pathways

## Abstract

Early-stage detection of leukemia is a critical determinant for successful treatment of the disease and can increase the survival rate of leukemia patients. The factors limiting the current screening approaches to leukemia include low sensitivity and specificity, high costs, and a low participation rate. An approach based on novel and innovative biomarkers with high accuracy from peripheral blood offers a comfortable and appealing alternative to patients, potentially leading to a higher participation rate.

Recently, non-coding RNAs due to their involvement in vital oncogenic processes such as differentiation, proliferation, migration, angiogenesis and apoptosis have attracted much attention as potential diagnostic and prognostic biomarkers in leukemia. Emerging lines of evidence have shown that the mutational spectrum and dysregulated expression of non-coding RNA genes are closely associated with the development and progression of various cancers, including leukemia. In this review, we highlight the expression and functional roles of different types of non-coding RNAs in leukemia and discuss their potential clinical applications as diagnostic or prognostic biomarkers and therapeutic targets.

## Introduction

Leukemia is a class of blood cancers characterized by an oligoclonal expansion of hematopoietic cells that infiltrate the bone marrow and can also invade the blood and other extramedullary tissues [1]. The proliferation of leukemic cells causes the expulsion of the normal hematopoietic cells and the loss of their functions, leading to severe symptoms, including thrombocytopenia, anemia, and immunodeficiency. Hematological cancers are ranked as the 11th common type of cancer and the 10th common cause of cancer-related death. More than 350,000 new leukemia cases and 265,000 leukemia deaths were estimated to have occurred in 2012 [2]. In the United States, leukemia accounts for approximately 4% of cancer-derived mortalities and 3.5% of all cancer cases. The incidence, mortality, and survival of leukemia depends on the diagnosis, prognosis, as well as natural history of neoplasms arising from the malignant transformation of hemopoietic stem cells or progenitor cells in the bone marrow [3].

Leukemia can be classified according to its progression pattern (acute or chronic) and affected lineage (lymphoid or myeloid). The four major subtypes are acute lymphoblastic leukemia (ALL), chronic lymphoblastic leukemia (CLL), acute myeloid leukemia (AML), and chronic myeloid leukemia (CML) [4, 5]. ALL is one of the most common types of malignancy in children worldwide [6], while the other subtypes are more common in adults. In all types of leukemia, the abnormal proliferation of bone marrow and blood cells interferes with the production of functionally healthy cells. Thus, anemia ensues in people with leukemia resulting in reduced ability to fight infections and clotting disorders. For most patients, the causes of leukemia and its subtypes are unclear partly due to diverse abnormalities and multiple risk factors. However, the genetic background interacting with environmental factors including exposure to high doses of radiation or carcinogenic agents, such as benzene; parental occupational exposures; and infections all contribute to a higher risk of developing leukemia [7].

The underlying molecular mechanisms mediating the pathophysiology of leukemia are not fully understood. Thus, deeper insights in the genetic basis of the disease and their influence on the progress of the disease and treatment response are crucial to discovering new prognostic markers and novel therapeutic targets that can open new doors in personalizing treatment. The focus of research for decades has been on the expression of messenger RNAs that code for proteins. Recently, there has been much research suggesting that protein-coding genes only cover a small proportion of the human transcriptome and that a more significant proportion of the human transcriptome (66%) is composed of long non-coding RNAs (long ncRNAs), antisense and micro RNAs (miRNAs), and pseudogenes [8]. Current evidence has shown that ncRNAs might act as a link between the genome and the environment by being an intricate player in the process of gene expression, contributing to the pathogenesis of various human diseases, including cancer. Several studies have documented the involvement of ncRNAs in differentiation, proliferation, and apoptosis of leukemic cells and their potential as a future prognostic biomarker.

In the current review, we discussed the characteristics and role of leukemia related non-coding RNAs. We provided a succinct overview of the current understanding of non-coding RNA expression patterns in different types of leukemia, the mechanisms that contribute to leukemia carcinogenesis, and their role in drug resistance. Deciphering the essential role of diverse non-coding RNAs may improvise the understanding of the underlying biological events, ultimately leading to the identification of novel therapeutic targets, opening new prospects for treatment, diagnosis, and prognostication of different types of leukemia.

## Non-coding RNA networks and leukemia

Currently, there is an overpowering proof showing that transcriptional, posttranscriptional and translational controls, mediated by different non-coding RNAs, apply necessary pleiotropic activities on various highlights of leukemia science. This has opened space for disclosure and portrayal of non-coding RNAs as biomarkers in leukemia and prompted several investigations in this field over the last 10 years. The full picture of these unusually communicating non-coding RNAs in leukemia is slowly developing [9–17]. The vital role and underlying molecular mechanisms of non-coding RNAs and their therapeutic potential in leukemia are outlined in Table 1.

**Table 1:** Roles of ncRNAs implicated in leukemia

Type of ncRNA	ncRNA	Type of leukemia	Expression in leukemia	Mechanism/target/pathway	References
miRNA	miR-194-5p	AML	Upregulated	inducing BCLAF1; BCL2-associated transcription factor 1 (BCLAF1)	[18]
miRNA	miR-103	AML	Upregulated	Blocking PI3K/AKT signal pathway by regulation of COP1	[19]
miRNA	miR-15a	CML-CP	Upregulated	Expression modulated by BCR–ABL is linked to CML progression and imatinib resistance	[20]
miRNA	miR-130b	CML-CP	Downregulated	Expression modulated by BCR–ABL is linked to CML progression and imatinib resistance	[20]
miRNA	miR-145	CML-CP	Upregulated	Expression modulated by BCR–ABL is linked to CML progression and imatinib resistance	[20]
miRNA	miR-16	CML-CP	Downregulated	Expression modulated by BCR–ABL is linked to CML progression and imatinib resistance	[20]
miRNA	miR-26a	CML-CP	Downregulated	Expression modulated by BCR–ABL is linked to CML progression and imatinib resistance	[20]
miRNA	miR-146a	CML-CP	Downregulated	Expression modulated by BCR–ABL is linked to CML progression and imatinib resistance	[20]
miRNA	miR-29c	CML-CP	Downregulated	Expression modulated by BCR–ABL is linked to CML progression and imatinib resistance	[20]
miRNA	miR-96	AML	Downregulated	Oncogene Metastasis-associated lung adenocarcinoma transcript 1 (MALAT1) knockdown inhibited proliferation, promoted apoptosis and enhanced Ara-C sensitivity in AML cells by upregulating miR-96	[21]
miRNA	miR-128b	ALL	Downregulated	downregulation of the MLL-AF4 chimeric fusion proteins MLL-AF4 and AF4-MLL that are generated by chromosomal translocation t(4;11)	[22]
miRNA	miR-34a	AML	Downregulated	TUG1 confers Adriamycin resistance in acute myeloid leukemia by epigenetically suppressing miR-34a expression via EZH2	[23, 24]
miRNA	miR-451a	CML	Downregulated	NR	[25]
miRNA	let-7b-5p	CML	Downregulated	NR	[25]
miRNA	hsa-miR-425	AML	Upregulated	Through calcium signaling pathway and natural killer cell mediated cytotoxicity.	[26]
miRNA	hsa-miR- 200c	AML	Downregulated	NR	[26, 27]
miRNA	hsa-mir-30a	CML	Downregulated	NR	[28]
miRNA	miRNA-155	ALL	Upregulated	NR	[29]
miRNA	miR-130a	CML	Downregulated	Functions as a tumor suppressor by inhibiting multiple anti-apoptosis proteins, including BCL-2, MCL-1 and XIAP.	[30]
miRNA	miR-125b	AML; ALL	Upregulated	microRNA125b promotes leukemia cell resistance to daunorubicin through inhibiting apoptosis	[31]
miRNA	miR-224	CML	Downregulated	miR-224, along with let-7i, regulate the proliferation and chemosensitivity of CML cells probably via targeting ST3GAL IV.	[32]
lncRNA	HOXA-AS2	AML	Upregulated	HOXA-AS2 negatively regulates the expression of miR-520c-3p in ADR cells. S100A4 was predicted as a downstream target of miR-520c-3p,	[33]
lncRNA	TUG1	AML	Upregulated	TUG1 confers Adriamycin resistance in acute myeloid leukemia by epigenetically suppressing miR-34a expression via EZH2	[23, 34]
lncRNA	RP11-342 M1.7	AML	Upregulated	Involved in neoplastic signaling pathways	[35]
lncRNA	CDCA4P3	AML	Upregulated	Involved in neoplastic signaling pathways	[35]
lncRNA	CES1P1	AML	Downregulated	Involved in neoplastic signaling pathways	[35]
lncRNA	AC008753.6	AML	Downregulated	Involved in neoplastic signaling pathways	[35]
lncRNA	RP11-573G6.10	AML	Downregulated	Involved in neoplastic signaling pathways	[35]
lncRNA	MEG3	CML	Downregulated	contributes to imatinib resistance through regulating miR-21	[36]
lncRNA	PANDAR	AML	Upregulated	NR	[37]
lncRNA	GAS5	AML	Upregulated	Via affecting hematopoietic reconstitution	[38]
lncRNA	UCA1	CML	Upregulated	UCA1acts as a ceRNA Against miR-16 in Chronic Myeloid Leukemia Cells	[39]
lncRNA	MALAT1	CML	Upregulated	MALAT1 promotes imatinib resistance of CML cells by targeting miR-328	[40]
lncRNA	UCA1	AML	Upregulated	knockdown of UCA1 plays a role in overcoming the chemoresistance of pediatric AML, by inhibiting glycolysis through regulating the miR-125a/HK2 pathway.	[41]
lncRNA	NONHSAT076891	APL	Upregulated	NR	[42]
lncRNA	CRNDE	AML	Upregulated	NR	[13]
lncRNA	LINC00899	AML	Upregulated	NR	[12]
lncRNA	HOTAIR	CML	Upregulated	Knockdown of HOTAIR expression downregulates MRP1 expression levels and reverses imatinib resistance via PI3K/Akt pathway.	[43]
lncRNA	IRAIN	AML	Downregulated	Interaction with chromatin DNA and involvement in the formation of an intrachromosomal promoter loop	[44]
lncRNA	CCDC26	AML	Upregulated	NR	[45]
lncRNA	KCNQ1OT1	AML	Upregulated	NR	[46]
lncRNA	NONHSAT027612.2	ALL	Upregulated	Through regulating immune response-associated pathways.	[47]
lncRNA	NONHSAT134556.2	ALL	Upregulated	Through regulating immune response-associated pathways.	[47]
lncRNA	LINP1	AML	Upregulated	Via HNF4alpha/AMPK/WNT5A signaling pathway	[48]
lncRNA	SNHG3	AML	Upregulated	SNHG3 elicits a growth-promoting function in AML via sponging miR-758-3p to regulate SRGN expression	[49]
lncRNA	LUNAR1	ALL	Downregulated	Proliferation of T cells	[50, 51]
lncRNA	T-ALL-R-LncR1	ALL	Upregulated	Regulate apoptosis by Par-4/THAP1 protein complex	[52]
lncRNA	HOTAIRM1	AML	Upregulated	Chromatin modification, myeloid differentiation	[53, 54]
lncRNA	PVT1	AML	Upregulated	Oncogene, induce proliferation and suppress Apoptosis	[55]
lncRNA	ANRIL	AML/ALL	Upregulated	Myeloblast proliferation	[56]
lncRNA	BGL3	CML	Upregulated	Apoptosis and DNA methylation	[57]
circRNA	f-circPR	AML	Upregulated	High proliferation, chemo resistance, Differential expression	[58]
circRNA	circ-PVT1	AML	Upregulated	Involved in the development of leukaemia (AML)	[59]
circRNA	circNPM1 75,001 (hsa_circ_0075001)	AML	Upregulated	NPM1/regulate myeloid differentiation though miR-181,	[60]
circRNA	circ-HIPK2	AML	Downregulated	Regulate differentiation though miR-124-3p	[61]
circRNA	circRNA-DLEU2	AML	Upregulated	Enhanced cell division, survival, and proliferation with suppressed apoptosis through miR-496/ PRKACB	[62]
circRNA	hsa_circ_0004277	AML	Downregulated	Act as prognostic factor for survival outcome in AML patients. Target multiple miRNAs and Genes miR-138-5p, miR-30c-1-3p, miR-892b, miR-571, miR-328-3p/SH3GL2, PPARGC1A, PIP4K2C, SH2B3, ZNF275, and ATP1B4	[63]
circRNA	circ-CBFB	CLL	Upregulated	regulating miR-607/FZD3/Wnt/beta-catenin pathway	[64]
circRNA	circ_0132266	CLL	Downregulated	circ_0132266 acts as a sponge of miR-337-3p and regulates its activity, resulting in a downstream change of target-gene PML, influencing cell viability.	[65]
circRNA	circPAN3	AML	Upregulated	circPAN3-miR-153-5p/miR-183-5p-XIAP axis; circPAN3 may facilitate AML drug resistance through regulating autophagy and influencing expression of apoptosis-related proteins	[66, 67]
circRNA	circ_0009910	AML	Upregulated	knockdown of circ_0009910 inhibited AML cell proliferation and induced apoptosis by acting as a sponge for miR-20a-5p	[68]
circRNA	circ_100053	CML	Upregulated	involved in imatinib resistance	[69]
circRNA	hsa_circ_0080145	CML	Upregulated	knockdown of hsa_circ_0080145 significantly suppressed CML cell proliferation thorugh acting as a sponge for miR-29b.	[70]
circRNA	circ-ANAPC7	AML	Upregulated	circ-ANAPC7 targets the MiR-181 Family	[71]
circRNA	hsa_circ_0004277	AML	Downregulated	Increasing level of hsa_circ_0004277 by chemotherapy was associated with successful AML treatment	[63]
circRNA	circBA9.3	CML	Upregulated	Chemoresistance, Oncogene, Induce cell proliferation and supressed apoptosis	[72]
siRNA	SKP2	AML	Upregulated	SKP2 inhibits the degradation of P27kip1 and down-regulates the expression of MRP	[73]

## Characteristics of non-coding RNA networks

**Fig. 1:**
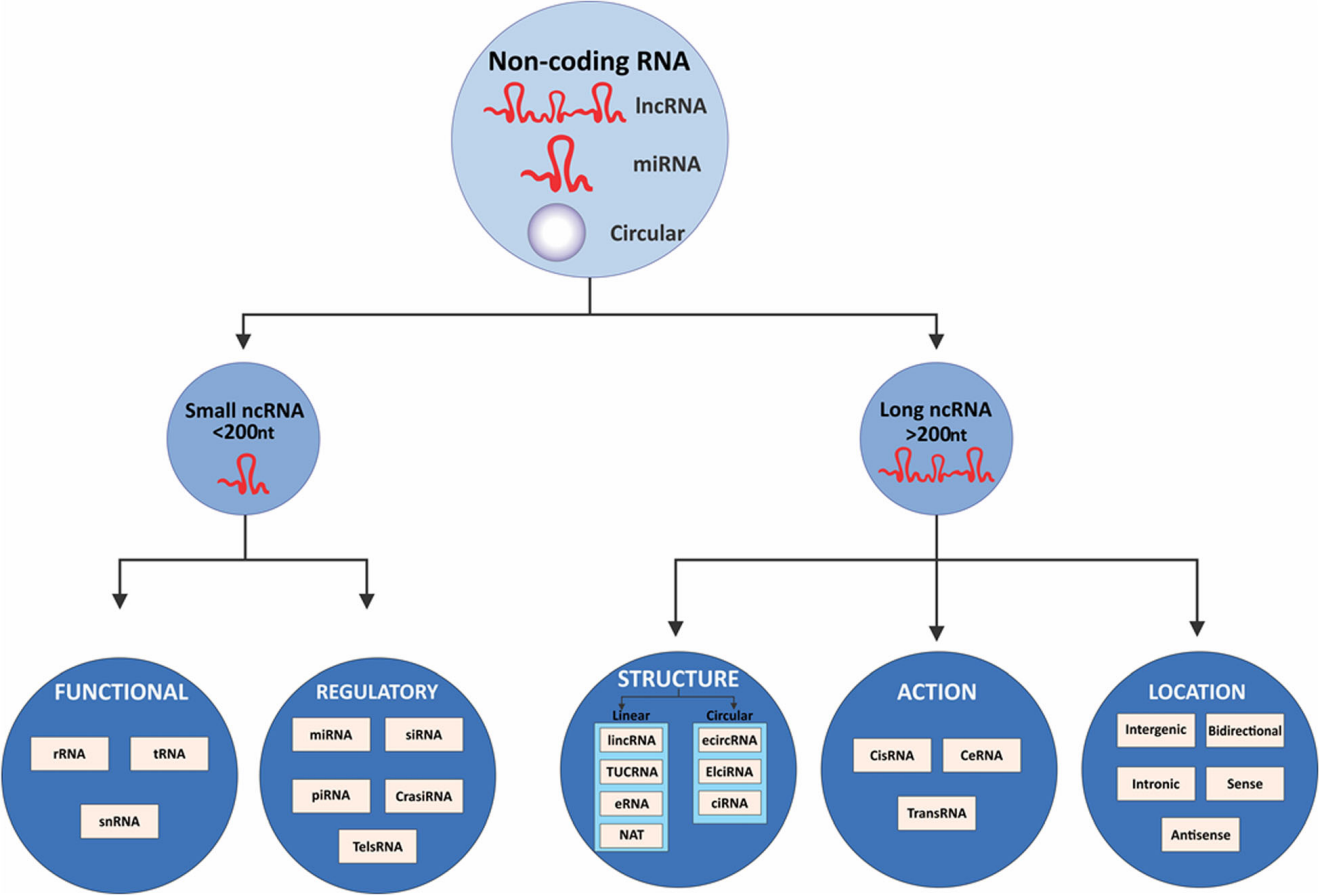
Classification of noncoding RNAs (ncRNAs). Noncoding RNAs are classified into small ncRNAs (< 200 nucleotides) or long ncRNAs (> 200 nucleotides) based on their length. Small ncRNAs are further classified into functional and regulatory noncoding RNAs while long ncRNAs are classified based on their structure, function and location

Latest proceedings in high-throughput sequencing for whole genomes and transcriptomes demonstrated that fewer than 2% of the entire human genome encodes proteins, whereas a large portion of the human genome, constituting at least 75%, encodes ncRNAs [74]. Currently, ncRNAs are classified according to transcript size into two broad categories, small (< 200 nucleotides; ncRNAs) and long (> 200 nucleotides; lncRNAs) non-coding RNAs (lncRNAs) **(**Fig. 1**)**. The ncRNAs play a major role in the process of gene expression, RNA maturation, and protein synthesis [75–77]. With the emerging evidence, it has become quite evident that not only protein-coding mutations but variations within the noncoding genome are also responsible for various cancer etiologies [78, 79].

## Long non-coding RNAs

**Fig. 2:**
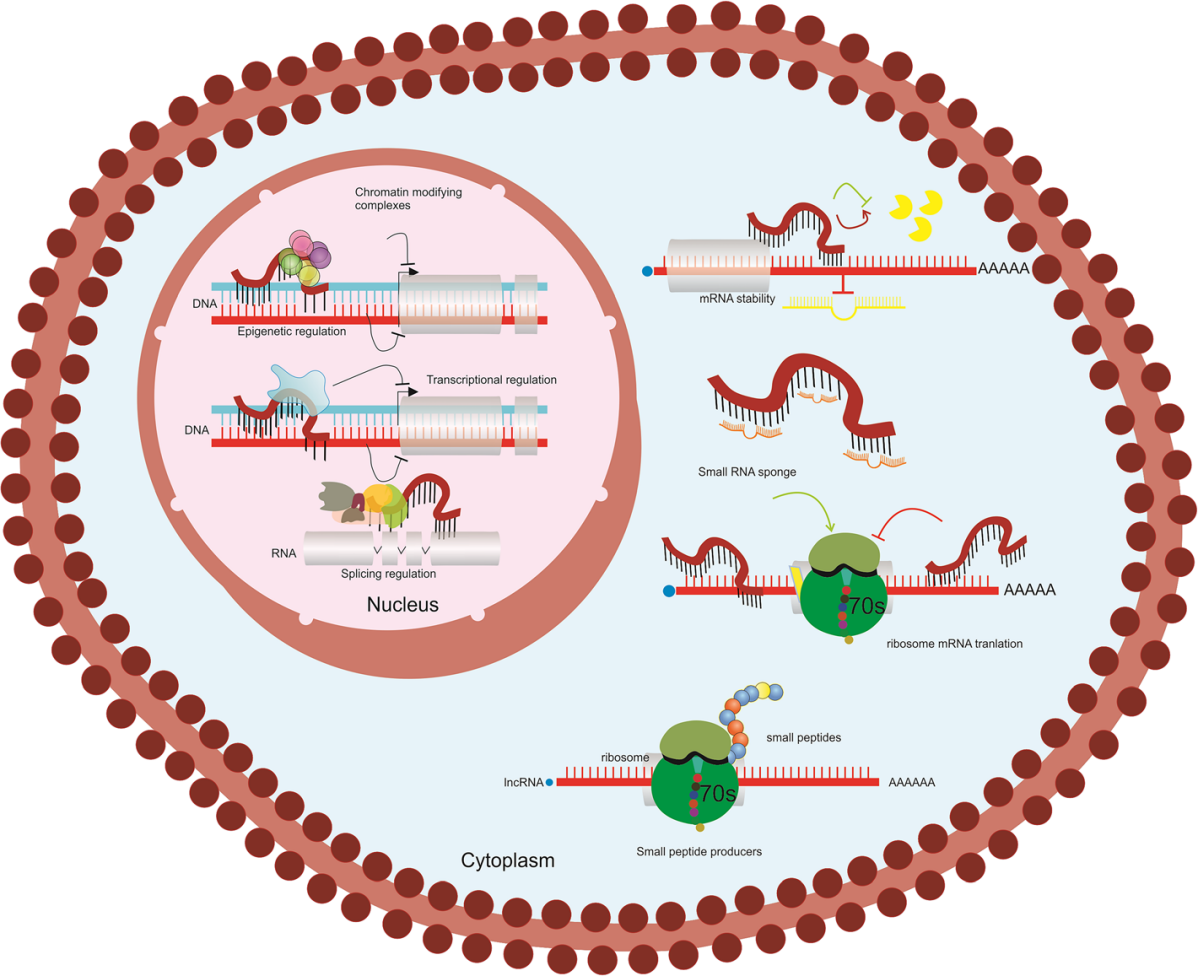
General mechanisms for functions of Long Noncoding RNAs. Nuclear lncRNAs are implicated in Epigenetic regulations, Transcriptional regulations, and splicing regulations while cytoplasmic lncRNAs are involved in mRNA stability, act as small regulatory RNA sponges, regulate mRNA translation and can also be small peptide producers

lncRNAs are defined as transcripts with lengths exceeding 200 nucleotides that are not translated into protein [80, 81], and most of them are markedly expressed in differentiated tissues or particular cancer types [78]. RNA polymerase II is responsible for executing the transcription of lncRNAs, and generally, they are expressed in a tissue-specific manner [78, 82]. LncRNAs regulate several biological processes such as differentiation, development and biogenesis and multiple human disorders, including certain malignancies are associated with deregulation of lncRNAs. Deregulation of lncRNAs was demonstrated to be intrinsically connected with human illnesses, including different kinds of malignant growths [78, 82]. Because of this, lncRNAs have become a focal point of researchers, and practical explanations of the roles of lncRNAs are an evolving line of research. Usually, lncRNAs utilize various instruments to implement their functions at a cellular level. For example, lncRNAs can influence chromatin redesigning and methylation, act as a miRNA restraint sponge, and regulate protein complexes stability [76, 83, 84] **(**Fig. 2**)**.

Several pieces of evidence have shown that some lncRNAs, for example, TARID, Kcnq1ot1, and AS1DHRS4, engage DNA methyltransferases to alter chromatin conformation or act to alter the position of nucleosome through the SWI/SNF complex as observed in SChLAP1 [85–87]. The histone methyltransferase poly-comb repressive complex-2 (PRC2) is a widely studied protein managed by ncRNAs and has shown as an intermediary target of lncRNAs [88]. PRC2 appears to play a role in inactivating chromatin through initiating the inhibitory H3K427me3 histone marks [88]. Also, chromatin alterations by specific lncRNAs, for example, HOTTIP and CCAT1, results in tweaking chromosome circling and influencing gene promoters [89, 90]. The lncRNA Firre was demonstrated to be crucial in maintaining inactivation of the X chromosome [91]. X-linked lncRNA Firre cohesion with the chromatin remodelers, CTCF and attachment, is one of the essential steps in the process and includes changing chromatin confirmation during the inactivation of X chromosome process. Subsequently, the inactive X chromosome is positioned close to the nucleolus and maintain H3K27me3 methylation [91]. Different lncRNAs have their distinct inhibitory roles regulated through the action of authoritative miRNAs, which can seize the biomolecules and diminish their potential to inhibit their targets [82].

The roles of miRNA in leukemia have been broadly explored in recent years, but the utilitarian roles of lncRNAs in such tumors are yet unclear. Numerous lncRNAs are deregulated in different sorts of malignant growths, including head and neck cancer [92]. Unmistakable expression profiles of lncRNA have been distinguished in leukemia [9, 33, 35, 45, 47–49, 93–99]. Some of these have been demonstrated to have well-understood jobs in the development and progression of leukemia, suggesting the vital use of lncRNAs as novel biomarkers and potential targets for the treatment of leukemia. Recent shreds of evidence have demonstrated that few lncRNAs play significant physiological roles and are essential for regulating different levels of gene expression [84, 100, 101]. While some of the lncRNAs act as oncogenes, others function as tumor suppressors, and they are involved in cellular processes, including the cell cycle and tumor invasion and metastasis [102]. For example, the lncRNA HOXA cluster antisense RNA2 (HOXA-AS2), which has been previously shown to have oncogenic properties in several human malignancies, was found to diminish glucocorticoid sensitivity in acute lymphoblastic leukemia through the HOXA3/EGFR/Ras/Raf/MEK/ERK pathway [33]. Likewise, exhaustive lncRNA expression profiling by RNA sequencing has uncovered that lncRNA RP11-342 M1.7, lncRNA CES1P1 and lncRNA AC008753.6 are both independent as well as in combination, serve as predictive factors for AML risk [35]. LncRNA LINP1 was found to regulate AML progression employing the HNF4alpha/AMPK/WNT5A signaling pathway [48]. miR-335-3p dysregulation, directed by the lncRNAs NEAT1 and MALAT1, is associated with a poor prognosis in childhood ALL. By and large, these discoveries provide a greater depth of understanding into the pathogenesis of a high-risk group of leukemias that can help clinicians explore the possibility of using lncRNAs for treatment.

## Micro RNAs

**Fig. 3:**
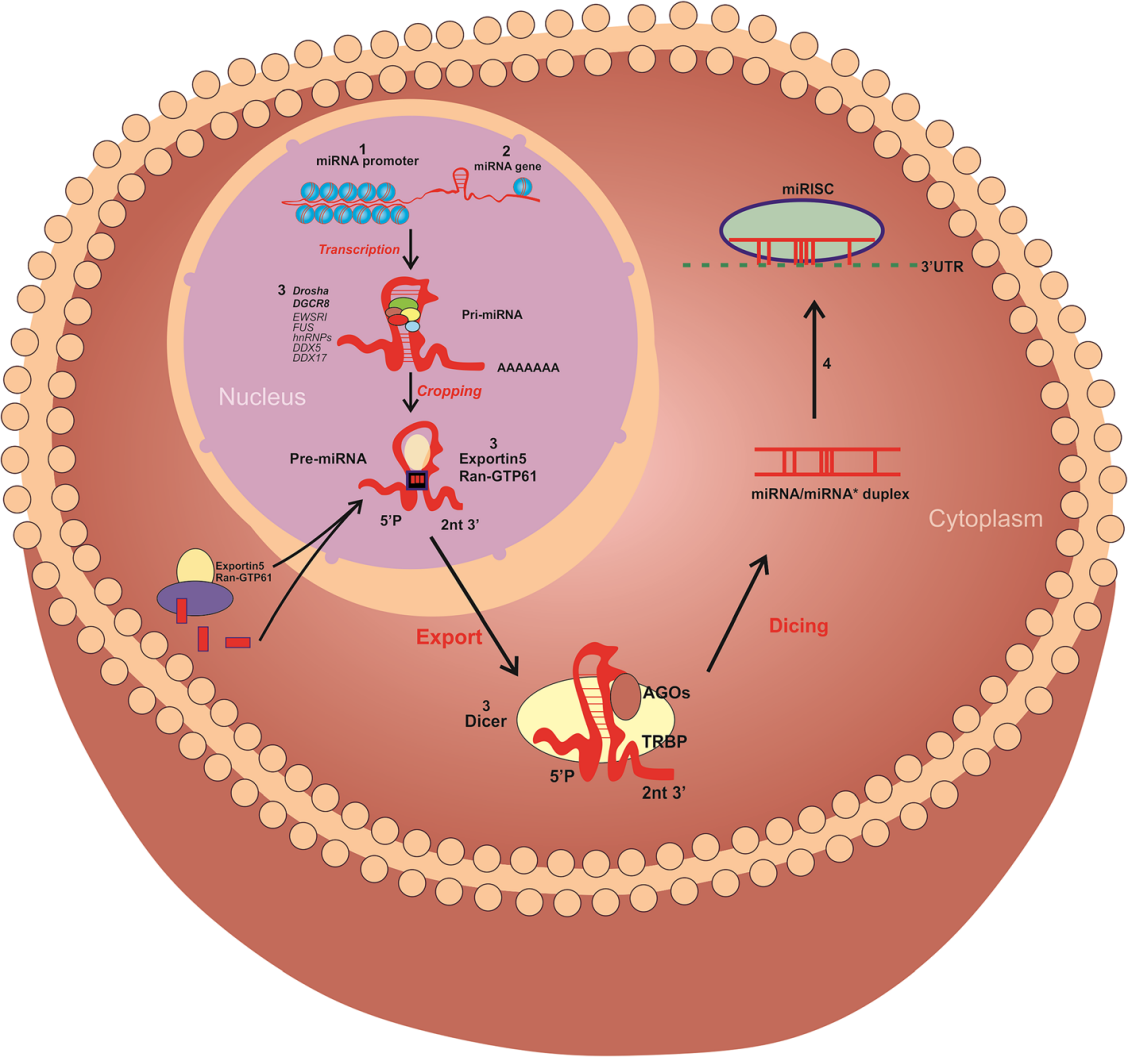
MicroRNA (miRNA) biogenesis and regulation of gene expression. The series of events includes the production of the primary miRNA (pri-miRNA) transcript by RNA polymerase II or III and cleavage of the pri-miRNA into a stem-loop structured miRNA precursor (pre-miRNA) by the microprocessor complex Drosha-DGCR8 (Pasha) in the nucleus. Then the pre-miRNA hairpin is exported from the nucleus by Exportin-5-Ran-GTP. In the cytoplasm, the RNase Dicer in complex with the double-stranded RNA-binding protein TRBP cleaves the pre-miRNA hairpin to its mature length. The functional strand of the mature miRNA is loaded together with Argonaute (Ago2) proteins into the RNA-induced silencing complex (RISC), where it guides the RISC to silence target mRNAs through mRNA cleavage, translational repression or deadenylation, whereas the passenger strand is degraded

Micro RNAs (miRNAs) are a subset of non-coding RNAs ~ 19–20 nt in length with 5′-phosphate and 3′-hydroxyl ends. The ribonuclease Dicer processes them from precursors having a characteristic hairpin secondary structure **(**Fig. 3**)**. miRNAs were first discovered in *Caenorhabditis elegans* and have since been found in most eukaryotes, including humans [103–105]. According to the reports, human genome comprised of approximately 1–5% of miRNA, which is responsible for at least 30% of the protein-coding genes [106–110]. To date, 940 distinct miRNA molecules have been identified [111–113]. The knowledge about the specific targets and biological functions of miRNA molecules is still scarce, but their crucial role in the regulation of gene expression, controlling diverse cellular and metabolic pathways is well-evident [114–119]. As this field is still emerging, there are only a limited number of studies in the context of miRNAs in leukemia.

While some of the miRNAs work as oncogenes, others work as tumor suppressors [120]. For instance, it has been shown that the balance between miR-194-5p and its target BCL2-associated transcription factor 1 (BCLAF1) is commonly deregulated in AML patients [18]. Also, miR-10a-5p was found to be overexpressed in relapsed AML cases [121]. Furthermore, the expression of miR-96 was downregulated in newly diagnosed AML and is associated with leukemic burden [122]. Collectively, these findings allow us to develop a better understanding of the underlying mechanisms of a high-risk group of leukemias that can assist clinicians in clarifying the function of miRNA and use this information to guide treatment.

## Role of microRNA gene abnormalities in leukemia

Abnormal expression of miRNA has been reported in many malignancies, including stomach [123], brain [124], breast [125], lung [126], liver [127], colon [128], leukemia [129] and lymphoma [130]. Many studies have reported that microRNA function as a tumor suppressor or oncogene. In most of the tumors, the tumor -suppressing miRNAs are downregulated, whereas the oncogenic miRNAs are overexpressed. Jongen-Lavrencic et al., [131] reported that *miR-155 is upregulated in* hematopoietic stem cells carrying *FLT3-ITD* and nucleophosmin *(NPM1)* gene mutations of AML patients. Similarly, Lagos-Quintana et al., [132] in murine lymphocyte precursors reported increased expression of miR-155 that induces polyclonal lymphocytosis and develops high-grade lymphocytic leukemia. Also, in the case of a myeloproliferative disease, the overexpression of miR-155 was reported that leads to increased granulocyte-monocyte cells [122]. Fuster O et al., [133] suggested that abnormal expression of miR-155 signaling targets SHIP1 and CEBPB in AML patients, both of which are critical in granulopoiesis. Yamamoto et al., [134] reported that miR-133 in leukemic cells targets the Ecotropic viral integration site 1 (Evi1) which upregulated the drug sensitivity and suggested that miR-133 can be a potential therapeutic target for Evi1-overexpressing leukemia. In AML cell lines, Xiao et al. [135] reported elevated expression of miR-223 that inhibited proliferation and cell motility but promote cell apoptosis. Several studies reported that ectopic miR-223 overexpression decreased the tumorigenesis by controlling the G1/S cell cycle phase transition [136]. Lin X et al., [137] investigated that the miR-370 expression was decreased in pediatric AML patients which in turn contribute to the significant progression of the disease and it was suggested that the miR-370 expression could act as non-invasive diagnostic and, a prognostic marker for pediatric AML patients. Magee P et al. has reported [138] that abnormal expression of microRNAs induce chemoresistance that affects a variety of cancer types and he also determined that the forced expression of miR-22 and miR-193a leads to inhibition of leukemia progression. Liu X et al., [139] conducted experiments in leukemic cell lines HL60, NB4, and K562 and reported that the upregulation of miR-181a induces higher cell proliferation thereby increased cell cycling by targeting ATM. It has been investigated that the transfection of miR-128 increased the drug sensitivity, enhanced apoptosis in HL60 cell lines [140], whereas the DNA damage was tolerated; however, the molecular mechanism is yet to be elucidated. However, Volinia S et al., [141] reported that miR-128 to be overexpressed and upregulated in different malignancies, but its expression was decreased in AML cells carrying NPM1 mutations. Imatinib Resistance has been reported as a major hurdle for the treatment of chronic myeloid leukemia (CML). The miRNAs are involved in various processes from the development to drug resistance of tumors, including chronic myeloid leukemia (CML). Recent data suggested that miR-221-STAT5 axis played crucial roles in controlling the sensitivity of CML cells to imatinib [142]. Another recent finding reports that lncRNA MALAT1/miR-328 axis promotes the proliferation and imatinib resistance of CML cells, providing new perspectives for the future study of MALAT1 as a therapeutic target for CML [40]. In addition, miR-214 was associated with the imatinib resistance in CML patients by regulating ABCB1 expression [143].miR-30e has been shown to be directly targeting ABL mRNA and leads to decreased translation of ABL protein [144]. In K562 cells, the increased expression of miRNA-30e induces apoptosis and suppresses proliferation and sensitized the cells to imatinib treatment. miR-203 enhances the sensitivity of CML patients to imatinib and its expression was downregulated in bone marrow of CML patients [145].

## Circular RNAs

Circular RNAs (circRNAs) are an abundant class of regulatory transcripts primarily derived from protein-coding exons and widely expressed across eukaryotic organisms, including *Homo sapiens* and *Mus musculus* [146–150]. They play an essential role in regulating gene expression [151] through forming covalently closed continuous loop structures with no exposed ends. CircRNAs are evolutionarily conserved, display a higher degree of relative stability in the cytoplasm and are often expressed in a tissue/developmental stage-specific trend [152]. Briefly, circRNAs are produced co-transcriptionally from precursor mRNA by back-splicing of RNA polymerase II transcribed genes and often expressed at only low levels. The biogenesis of circRNAs is regulated through cis and trans-acting regulatory elements that control splicing [153]. The structural form of most circRNAs is composed of multiple exons, and multiple circRNA isoforms can be expressed from a gene with the inclusion or exclusion of internal introns through alternative splicing [153–155].

**Fig. 4:**
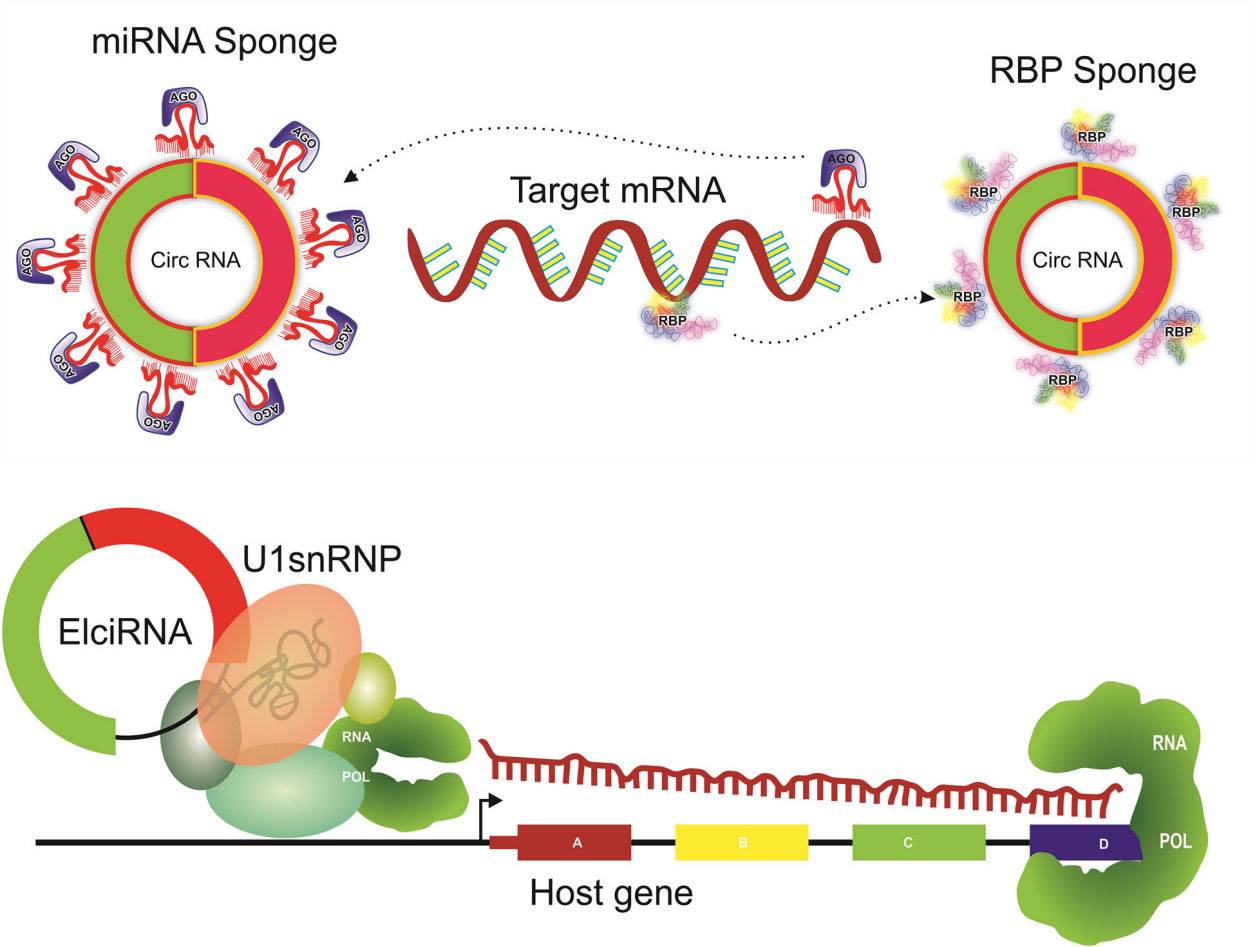
General mechanisms for functions of circular RNAs (circRNAs). circRNAs can function as a sponge for a miRNA/RBP keeping miRNA/RBP away (dashed arrows) from its mRNA targets, thus altering gene expression. Through interaction with U1 snRNP, exon-intron circRNAs (EIciRNAs) can interact with transcription complexes at host genes to induce their transcription

Recent studies have shown that several circRNAs play important physiological and functional roles at multiple stages of the gene expression regulation cascade [84, 100, 101]. CircRNAs are known to be involved in post-transcriptional regulation by acting as decoys for binding of micro RNAs, reducing their cellular availability and resulting in the upregulation of their target mRNAs **(**Fig. 4**)**. For example, circRNA ciRS-7, also known as CDR1as, is produced from the vertebrate cerebellar degeneration-related 1 (CDR1) antisense transcript and acts as an RNA sponge to repress miR-7 activity [148, 156]. Knockout mice of CDR1as show defects in sensorimotor gating [157] and knockdown of CDR1as expression results in a decrease of tumor growth and proliferation in cancer cell lines [158, 159]. Another circular RNA produced from the Sry gene has 16 binding sites for miR-138, and overexpression constructs of Sry circRNA attenuate the knockdown effects of miR-138 target mRNAs [156]. Indeed, multiple studies have remarkably demonstrated the potent sequestering effects of miRNA activity by circRNAs, making them excellent agents for competing endogenous RNA activity [148, 156, 160–164]. Increasing evidence also suggests that circular RNAs could perform other functional roles such as storage or sequestration of transcription factors and RNA binding proteins [165], microRNA transport [157] or encode functional proteins [166–169].

CircRNAs are altered in a variety of pathological conditions, which has stimulated significant interest in their role in human disease and cancer. There is emerging evidence that circRNAs show close association with many human diseases, including cancers – often but not always involving micro RNA (miRNA) intermediate. One study revealed hundreds of circRNAs being more abundant in blood than corresponding linear mRNAs, which suggests that circRNAs could be used as new biomarkers in standard clinical blood samples [170]. For instance, circ-CBFB was found to promote proliferation and inhibit apoptosis in CML by regulating the miR-607/FZD3/Wnt/beta-catenin pathway [171]. Additionally, circ_0009910 was found to be significantly upregulated in AML patients, and its high expression was shown to be associated with poor outcomes of AML patients [68]. Similarly, hsa_circ_0080145 was found to regulate CML cell proliferation by acting as a miR-29b sponge, and its knockdown was found to suppress CML cell proliferation [170] significantly. On the other hand, circRNAs circ_0132266 and hsa_circ_0004277 were found to be significantly downregulated in CLL and AML, respectively [63, 65].

We have identified multiple circular RNAs that are differentially expressed in metastatic versus primary ovarian tumors [172]. These circRNAs exhibits a robust expression pattern compared to their linear counterparts with higher power to distinguish between tumor subtypes. This may offer a more robust diagnostic marker of disease progression and prognosis. Our new results have indicated a substantial genetic control of the circular RNA expression that is mostly independent of the basal gene expression [173]. The power to distinguish between tumor subtypes along with an independent genetic control mechanism for their expression strongly points towards a functional and regulatory role for the circular RNA structures and their potential to contribute to disease pathogenicity. It is, therefore, worthwhile to investigate the mechanisms for biogenesis of circRNAs and their contribution to pathogenesis; this may lead to the development of new therapeutic interventions and biomarkers with diagnostic and prognostic capabilities.

## Underlying mechanisms of chemoresistance regulated by ncRNAs in leukemia

As in many cancers, resistance to therapy is a significant problem in the treatment of leukemia patients. The most commonly used chemotherapeutic drugs like bendamustine, chlorambucil, and rituximab [174, 175] although show initial response, but later on patients acquire resistance to these therapeutic regimens, hence limiting their efficacy. Also, many leukemia patients show resistance before treatment (intrinsic resistance) and therefore do not even show initial response. While the molecular mechanisms for both intrinsic and acquired resistance are mostly unidentified, identification of unique targets and pathways involved are still an area of intense investigation. Though genetic and epigenetic modifications that result in dysregulation of multi drugs transporters, alterations of drug targets & metabolism of drugs, defects in apoptosis & autophagy machinery, disruption of redox system, increased DNA repair and increased stem cell populations. Have been identified as mediators of drug resistance, the exact mechanisms of drug resistance, cross-talk among different mechanisms and their regulation are still under investigation. Recently, studies have conclusively established the role of miRNAs in chemotherapeutic resistance in leukemia [176, 177]. These studies have shown the deregulation of many miRNAs and their association with resistance to chemotherapy. For example, miR-181a and miR-181b are downregulated in chronic lymphocytic leukemia (CLL) [138] and overexpression of these miRNAs sensitize CLL cells to fludarabine mediated cell death by targeting B-cell lymphoma − 2 (BCL − 2), myeloid cell leukemia-1 (MCL-1) and X-linked inhibitor of apoptosis protein (XIAP) [178]. Similarly, restoration of miR-181b sensitize leukemia cells to doxorubicin (DOX) and cytarabine (ara-C) by downregulating MCL-1 and high mobility group box-1 (HMGB1) expression [179]. On the contrary, ectopic overexpression of miR-125b in leukemia cells induced resistance to daunorubicin (DNR) and prevented apoptosis by downregulating G-protein-coupled receptor kinase 2 (GRK2) and p53 -upregulated modulator of apoptosis (PUMA) [180].

Chronic myeloid leukocyte (CML) is characterized by the Philadelphia (Ph) chromosome [181] with fusion protein breakpoint cluster region-Abelson murine leukemia (BCR-ABL) tyrosine kinase overexpression. Interestingly, Imatinib, an inhibitor of BCR-ABL, show improved therapeutic efficacy in Ph-positive CML patients [182]. Interestingly, downregulation of ABL targeting miRNA-30e was reported in CML cell lines and patient samples [182]. Furthermore, overexpression of miRNA-30e in K562 leukemia cells suppressed proliferation, induced apoptosis and sensitized them to Imatinib treatment. While miRNA-203 sensitizes CML cells to Imanitib and induces apoptosis [145], miRNA-486, on the other hand, promotes Imanitib resistance by targeting PTEN and FOXO1 [183].

While the intrinsic resistance is due to many factors, including miRNA in our case, acquired resistance by tumor cells is promoted in response to continuous drug treatment. DNR and ara-C (anthracyclines) are most commonly used and effective chemotherapeutic drugs for leukemia treatment [184]. Though the use of these drugs results in the complete remission of the disease, most of the patients relapse within 5 years [185, 186], while inefficient tumor cell targeting, mutagenic effects of the drug or selection of resistant clones might be the reasons for relapse and development of aggressive tumors, however the underlying mechanism(s) are still to be identified. These anthracyclines by intercalating into the DNA and targeting Topoisomerase II [187, 188] hinder replication [189]. Interestingly, Topoisomerase II is downregulated in drug-resistant AML subtypes [190, 191], thus making these tumors resistant to these drugs. The topoisomerase II cuts DNA strands and binds to the scaffold/matrix-associated protein region (S/MAR) to prevent or resolve DNA supercoils. Therefore, anthracycline treatment results in DNA double-strand breaks which can be temporarily fixed by non-homologous end joining leading to gene mutation and t4:11 is a common mutation that occurs at S/MAR in AML [192–194]. S/MARs by interacting with HDACs regulate expression of miRNAs like miR-93, miR-221, miR-17, let-7b and miR-17-92 cluster. While the dislocation or loss of S/MAR can modulate miRNAs expression [195], anthracyclines like daunorubicin can induce DNA damage associated with deregulation of miRNA expression in leukemia.

Though anthracyclines by modulating miRNA expression regulate cell proliferation and apoptosis, specific miRNAs modulate the DNA repair signaling pathway components resulting in the development of therapeutic resistance. In this connection, resistance to daunorubicin (DNR) has been associated with overexpression of miRNA-21 and its downregulation in resistant K562/DNR cells enhanced DNR cytotoxicity in vitro. Similarly, overexpression of miR-181a in HL60, NB4, and K562 cells by targeting ataxia telangiectasia mutated (ATM) increased proliferation [139]. Also, miR-128 by targeting Rad51 promoted DNA damage and sensitized AML OCI-AML3 and MV4–11 cells to oral nucleoside analog prodrug called sapacitabine [196]. Though upregulated in many cancers, miR-128 is downregulated in AML, especially carrying NPM1 mutations [141, 197]. However, ectopic overexpression of miR-128 in HL60 cells increased drug sensitivity and promoted apoptosis [140]. In addition to miR-128, other miRNAs such as miR-103, miR-107, and miR-506 have been found to target Rad51 in other cancers as well. More specifically, miRNA-125b is overexpressed in pediatric acute promyelocytic leukemia (APL) than in other subtypes of acute myelogenous leukemia (AML), and its exogenous expression in AML cells imparted DOX resistance [198].

ABC transporters are most important proteins promoting drug resistance in almost all the tumors. While the above mentioned miRNAs impart drug resistance, many other miRNAs that are involved in sensitizing cancer cells to therapeutic drugs by targeting ABC transporters are downregulated in cancer [199]. In this category, miR-326 was found to downregulate the ABC transporter ABCC144 in resistant HepG2 cells and sensitize them to chemotherapeutic drugs. In addition to ABCC144, miR-326 also negatively regulated other ABC family members such as ABCA2 and ABCA3, which are drug-resistance related genes [200]. However, the miR-326 expression is reported to be significantly downregulated in the multidrug resistance (MDR+) pediatric ALL patients compared to the (MRD-) group [27]. A recent study showed upregulation of miR-125b-2 cluster (Let-7c, miR-125b, and miR-99a) in leukemia patients with ETV6-RUNX1^**+**^ fusion gene expression.

Further studies showed that knockdown of miR-125b in REH ETV6-RUNX1^**+**^ cells result in increased sensitivity to staurosporine and doxorubicin treatment, while overexpression of miR-125b-2 cluster inhibited apoptosis and increased cell survival suggesting its therapeutic potential in pediatric ALL [201]. In a recent comprehensive study, the involvement of miRNAs in L-asparaginase (L-ASP), vincristine (VCR), prednisolone (PRED) and DNR resistance was investigated [202]. This study showed the involvement of miR-454 in resistance to L-ASP, miR-125b, miR-99a, & miR-100 to DNR and miR-125b to VCR resistance. Furthermore, over expression of miR-125b prevented VCR mediated apoptosis in vitro [202]*.* Interestingly, leukemia ETV6-RUNX1+ patients with high expression of miR-125b show resistance to VCR treatment. Like chemotherapeutic drugs, use of glucocorticoids (GCs) for clinical treatment of pediatric ALL is also limited by the development of resistance resulting in poor patient response. Involvement of miRNAs in resistance/sensitivity to GC treatment has recently been evaluated [203]. In a genome-wide study, while the expression of miR-335 was found to be downregulated in all pediatric ALL patients, its overexpression sensitized ALL cells to PRED treatment in vitro [204]. In addition to PRED, ALL cells with miR-335 overexpression showed resistance to other chemotherapeutic drugs with limited cell death [204]. Another miRNA, miR-210 is differentially expressed in various types of cancers including leukemia [205]. Using agomiR or antagomiR for miR-210 in LEH cells (to either increase or decrease the expression respectively) modulated the response to dexamethasone (DEX), L-ASP, VCR and DNR [205], suggesting that use of agomiR’s/antagomiR’s can be a novel alternative to overcome miRNA mediated therapeutic resistance in cancers including leukemia [205].

## Role of non-coding RNAs in immune modulation in leukemia

Several ncRNAs, including miRNAs, lncRNAs and circRNAs have been implicated in the modulation of the immune system in various human malignancies, including leukemia. These ncRNAs can modulate immune system either directly by regulating the differentiation of immune cells or indirectly by regulating the expression of various signaling molecules, including NF-kB, c-Myc, p53 and Notch. In this section, we will discuss the available evidence on the role of ncRNAs in immune modulation and its implications in leukemia. Most leukemia are driven by genetic or epigenetic abnormalities in hematopoietic stem cells (HSCs) or progenitor cells, leading to differentiation arrest and increased proliferation and survival of immature blasts in the bone marrow. In one of the first studies on understanding the role of lncRNAs in early hematopoietic differentiation, RNA sequencing of HSCs led to the identification of two lncRNAs, lncHSC-1 and lncHSC-2 [206]. Their depletion resulted in altered myeloid differentiation, impaired self-renewal of HSCs and increased T cell differentiation [206]. These results indicate that lncRNAs can regulate HSC differentiation, and any deregulation in their expression might contribute to various hematological malignancies by altering the differentiation of various HSCs. Indeed, several ncRNAs have been found to contribute to leukemogenesis through immune modulation and altering cell differentiation. HOXA transcript antisense RNA, myeloid-specific 1 (HOTAIRM1) is a myeloid-specific long intergenic non-coding RNA (lincRNA), and it is upregulated during myeloid maturation [207]. Knockdown of HOTAIRM1 in the human acute promyelocytic leukemia (APL) cell line NB4 resulted in decreased granulocytic maturation [53]. HOTAIRM1 is known to regulate the expression of the HOX, CD11b and CD18 genes, which are required for myeloid cell differentiation [53]. Pathway analysis of HOTAIRM1 knockdown NB4 cells treated or untreated with all-trans retinoic acid (ATRA) revealed significant alterations in leukocyte mediated immunity, MHC class I protein complex, complement control module and regulation of leukocyte activation pathways [53]. Furthermore, HOTAIRM1 expression is also modulated by another transcription factor, PU.1, during granulocyte differentiation [208]. PU.1 is a master regulator of myeloid differentiation, while PU.1, along with IRF8, is known to control the fates of follicular (FO) and germinal centers (GO) B cells [209]. Double knockout of IRF8 and PU.1 in B cells has been shown to impair the development of FO and GC B cells [209]. This signifies that HOTAIRM1 can modulate tumor immunity in leukemia by interacting with other regulatory molecules. PU.1 is also known to drive the expression of lnc-DC, which is a lncRNA exclusively expressed in human dendritic cells (DCs) and is required for the differentiation of DCs [210]. Knockdown of lnc-DC resulted in impaired DC differentiation and function, and these effects were mediated by lnc-DC by regulating the posttranslational modification of a critical DC transcription factor, STAT3 [210]. Some of the proteins found to be altered after lnc-DC knockdown include those involved in antigen presentation (HLA-DR), cytokine secretion (IL-12) and T cell activation (CD40, CD80, and CD86). PU.1 also induces miR-23-27-24 cluster and plays a vital role in the regulation of immune cell lineage commitment [211].

Furthermore, this miRNA cluster regulates lymphoid cell differentiation and promotes myeloid lineage commitment and cell proliferation by directly targeting various lymphoid transcription factors, including Runx1 [211]. A recent study has identified a lincRNA, LINC00173, to be very specifically expressed in mature granulocytes [212]. Knockdown of LINC00173 in human CD34+ HSCs resulted in a defect in granulocytic differentiation and an increase in myeloid precursors in vitro [212]. Depletion of LINC00173 in NB4 leukemia cells, which carry an intrinsic block of granulocytic differentiation, resulted in reduced cell proliferation, signifying its role in early myelopoiesis [212]. Functional studies revealed the binding of LINC00173 with the EZH2 subunit of PRC2 [212]. X-inactive specific transcript (Xist) is another lncRNA reported in various human malignancies, including leukemia. Conditional knockout of Xist in murine hematopoietic cells resulted in myeloid leukemia and other impairments such as bone marrow dysfunction, lymphoid organomegaly and lymphoid infiltration of end organs [213]. Aforementioned examples emphasize the importance of ncRNAs in regulating immune cell differentiation, which is of great clinical relevance in leukemia.

The tumor suppressor p53 is known to induce the expression of lncRNA activator of enhancer domains (LED) in cancer [214]. The expression of LED is downregulated in leukemia, possibly due to promoter hypermethylation [214]. Another lncRNA, encoded from the first intron of the human p53 gene and known as lncRNAp53int1, is shown to be highly expressed in undifferentiated human myeloid leukemia cells [215]. However, expression of lncRNAp53int1 is significantly reduced during terminal differentiation of human leukemia cells into monocytes and macrophages [215]. Since several drugs have been used to induce differentiation of leukemia cells, targeting of lncRNAp53int1 could offer a newer therapeutic approach for the management of leukemias. Induction of p53 has also been shown to induce two other lncRNAs, nuclear enriched abundant transcript 1 (NEAT1) and lincRNA-p21, in primary human CLL [216]. The expression of NEAT1 is downregulated and seems to be regulated by PML-RARα in APL [217]. NEAT1 is also found to regulate myeloid differentiation in APL [217]. Recently, pharmacological activation of p53 has been shown to induce an immune-inflammatory response by activating NK cells, leading to suppression of leukemia growth [218]. However, p53 activation also results in the overexpression of PD-L1 in the surviving leukemia cells, promoting their immune escape [218]. All these evidences suggest a crucial role of p53 in regulating lncRNAs during immune modulation in leukemia.

Enhancer RNAs (eRNAs) are another class of lncRNAs and have been reported to be involved in immune modulation. Brazao et al. identified three lncRNA loci (LNCGme00432, LNCGme00344 and LNCGme00345), all of which are eRNAs, in a mouse model of B-ALL [219]. All of these eRNAs interact with PAX5, a transcription factor required for B-cell development and associated with the development of B-ALL, and are downstream of the B-cell lymphoma 11a (Bcl11a) gene [219]. Since the Bcl11a gene is required for VDJ recombination of immunoglobin genes and is also involved in B-cell development, a role of these eRNAs along with the PAX5 and Bcl11a genes in normal B-cell development and immune modulation in B-ALL cannot be ruled out.

In CLL, more than 50% of cases carry a deletion of the critical region at 13q14.3 [220, 221]. In addition to various tumor suppressor genes, miR-15a/16–1 and lncRNAs, deleted in lymphocytic leukemia 1 (DLEU1) and 2 (DLEU2), are also transcribed from this locus [222]. The miRNAs and lncRNAs have been reported to be deleted and epigenetically regulated in CLL [222, 223]. Interestingly, DLEU1 and DLEU2 are also known to regulate NF-kB activity through other NF-kβ regulating genes. Furthermore, the miR-15/16 family of genes is also known to induce NF-kβ activity [222] strongly. In CLL, NF-kB signaling is reported to be active, usually through interaction with the tumor microenvironment (TME), which leads to the survival of leukemia cells [224]. Another lncRNA, p50-associated COX-2, extragenic RNA (PACER), which is transcribed from the upstream region of the human COX-2 gene, regulates COX-2 expression by interacting with the repressive p50 subunit of NF-kβ, thereby functioning as a decoy lncRNA for NF-kB signaling [225]. NF-kB induced lncRNA, linc-Cox2, coactivates NF-kB, leading to induction of late-primary response genes in innate immune cells [226]. Since the NF-kβ family of transcription factors plays a crucial role in the regulation of tumor inflammation and immunity [227], we suggest that the NF-kβ as mentioned above regulated ncRNAs might also modulate immune system in leukemia.

Notch-regulated oncogenic lncRNA, leukemia-induced non-coding activator RNA-1 (LUNAR1), has been identified in T-cell acute lymphoblastic leukemia (T-ALL) [228]. Mechanistically, LUNAR1 regulates IGF signaling and induces IGF1R expression, leading to the survival of T-ALL cells [228]. The expression of LUNAR1 is upregulated in primary T-ALL cells, more so in Notch mutated samples, whereas its expression is suppressed upon Notch inhibition [228]. Another lncRNA, NOTCH1 associated lncRNA in T ALL (NALT), is also found to be associated with the Notch1 gene and functions as a transcription factor to activate Notch signaling and promote cell proliferation in pediatric T-ALL cells [229]. Role of Notch signaling in normal and effector immune cell differentiation is well established [230]. Furthermore, Notch can regulate various components of TME, including immune cells, fibroblasts, endothelial, and mesenchymal cells [230]. Since Notch signaling is also involved in human T-ALL [228, 229], we believe that Notch-regulated lncRNAs can potentially modulate immune system in leukemia.

Beta Globin Locus 3 (BGL3) is a lncRNA that regulates Bcr-Abl mediated cellular transformation in CML [57]. Bcr-Abl has been found to negatively regulated BGL3 expression through c-Myc-dependent DNA methylation in CML [57]. Interestingly, BGL3 acts as a competitive endogenous RNA (ceRNA), and it is targeted by many PTEN regulating miRNAs, including miR-17, miR-93, miR-20a, miR-20b, miR-106a and miR-106b [57]. It is well known that loss of PTEN in cancer cells leads to an immunosuppressive microenvironment through secretion of various immunosuppressive cytokines, recruitment of myeloid-derive suppressor cells (MDSCs) and regulatory T-cells (Tregs), and inhibition of CD8+ T-cell killing [231]. Hence, we speculate that BGL3 might also lead to immune modulation in leukemia through PTEN and PTEN-regulating miRNAs, although this needs to be experimentally proven. Colon cancer-associated transcript-1 (CCAT1) is a lncRNA that is known to be highly expressed in adult AML [153]. CCAT1 represses monocytic differentiation and promotes leukemia cell growth by upregulating oncogenic c-Myc and suppressing tumor suppressive miR-155 [153]. c-Myc is also known to induce lncRNA H19 expression in leukemia cells, thereby promoting cell proliferation and survival [232]. *Plasmacytoma* variant translocation 1 (PVT1) is another lncRNA that exerts its oncogenic effects by stabilizing the c-Myc protein in cancer [233]. Furthermore, in leukemia and other solid tumors, c-Myc is known to induce the expression of cluster of differentiation 47 (CD47), an innate immune regulator, and programmed death-ligand 1 (PD-L1), an adaptive immune checkpoint protein, involved in suppressing the antitumor immune response [234]. Hence, we believe that lncRNAs regulated by c-Myc might also modulate the immune response in leukemia.

Recent evidence also suggests a crucial role of circRNAs in immune modulation and leukemia development. The presence of fusion circRNAs (F-circRNAs) has been shown in PML/RARα positive APL and MLL/AF9 positive AML cells [58]. These F-circRNAs not only caused cellular transformation by activating PI3K and MAPK signaling but also contributed to leukemia cell proliferation, survival, progression and therapy resistance in vivo [58]. Since immune cells also regulates cell proliferation, survival and confer resistance to therapy, we believe that oncogenic F-circRNAs might also be involved in modulating the host immune system in leukemia, giving a survival advantage to leukemia cells. Because the presence of circRNAs has also been detected in extracellular vesicles [91], these circRNAs may modulate TME through cell-to-cell communication, although this is yet to be experimentally proven. Another circRNA, hsa_circ_0075001, has been detected in AML where its expression positively correlated with total NPM1 expression [60]. AML patients carrying a high expression of hsa_circ_0075001 had lower expression of components of the Toll-like receptor signaling pathway, suggesting that this circRNA might be involved in the modulation of the immune response in AML [60]. Another circRNA, circMYBL2, which is derived from the cell-cycle checkpoint gene MYBL2, has been reported to be highly expressed in FLT3-ITD mutation-positive AML patients [235]. Depletion of circMYBL2 inhibited proliferation and induced differentiation of FLT3-ITD AML cells in vitro and in vivo [235]. In a recent study of a comprehensive analysis of circRNA expression during hematopoiesis, the expression of circRNA was found to be highly cell-type specific during hematopoietic differentiation [236]. All these studies highlight the crucial role of circRNAs in immune modulation in leukemias.

Several miRNAs have been shown to modulate immune checkpoint proteins in various human malignancies, including leukemia. In AML, miR-34 regulates PD-L1 expression by targeting PD-L1 mRNA, thereby controlling PD-L1 specific T-cell apoptosis of human AML cells [85]. The miR-17-92 cluster, which encodes six miRNAs including 17, 18a, 19a, 20a, 19b-1, and 92–1, is also known to regulate T-cell responses in graft-versus-host disease (GVHD) post allogeneic bone marrow transplantation in mice [237]. This miRNA cluster has been found to promote CD4 T-cell activation, expansion, migration and Th1 differentiation while suppressing Th2 and Treg differentiation. Inhibition of miR-17 or miR-19b significantly inhibited alloreactive T-cell expansion and IFN-γ secretion, leading to prolonged survival in recipient mice with GVHD while preserving the graft-versus-leukemia effect [237]. Overexpression of miR-125a-5p has been shown to induce granulocytic differentiation, whereas miR-17-92 has the opposite effect in APL cells [238]. A recent study has identified overexpression of miR-708 in AML patients, which delayed HOXA9 mediated transformation in vivo by modulating myeloid differentiation [239]. The authors concluded that miR-708 is an indirect regulator of the HOX program during normal and impaired hematopoiesis [239].

## Clinical significance of ncRNAs in leukemia

In the current exploratory genomic era, the cellular or extracellular level of noncoding RNAs (ncRNAs) are advancing for their roles in risk stratification, diagnosis, and prognosis. Biologically ncRNAs regulate different processes such as proliferation, apoptosis, stemness, and differentiation. The clinical significance of ncRNAs in leukemia broadly illustrates their capability for risk stratification, diagnosis, and prognosis [212, 240, 241]. The quantitative assessments of transcripts by highly sensitive assay (qPCR) for minimal residual disease detection make ncRNA as a suitable candidate biomarker. The residual transcript copies play a significant role in detecting minimal residual disease. The best analogy is BCR-ABL international scale detection for deep molecular and ultra-deep molecular response in Philadelphia positive leukemias.

The prerequisite for ncRNAs as biomarkers in leukemia is their aberrant expression in leukemic phenotype., A plethora of differential miRNA, lncRNA and circRNAs from high throughput data, supported the notion and met this primary concern. However, leukemia itself is a disease of heterogeneous cell population; therefore, precisely identifying the robust biomarker in variable data sets of different leukemia subtype is very challenging at the validation step. Furthermore, the ncRNA fine-tune the cellular homeostasis; therefore their regulatory function activated with a slight change in the oncogenic molecular thrust. The ncRNAs modulates and attempt to reconcile the abnormal molecular changes. Recently, three-lncRNA expression-based risk score was developed based on RNA-seq data for AML patients using two leading data repositories [Therapeutically Available Research to Generate Effective Treatments (TARGET) and The Cancer Genome Atlas (TCGA)]. According to prognosis modelling, which was developed based on survival data, the combination of the lncRNA risk score and cytogenetics risk group provided a higher prognostic value than any of the individual prognostic factor [61].

Acute myeloid leukemia is a heterogenous malignancy of defective stem cells with impaired proliferation and differentiation. Many regulatory ncRNAs largely regulate the deregulation, stemness, proliferation and differentiation. Various studies have proved that many deregulated miRNAs are correlated with acute leukemia as compared to control samples. Table 1 shows a list of significant ncRNAs (lncRNA and circRNAs) for their pathological and clinical significance in leukemia.

HOTAIRM1 is located between HOXA1 and HOXA2 gene cluster and regulate granulocytic differentiation in hematopoiesis. High HOTAIRM1 expression results in increased expression of HOXA4 gene expression and defective myelopoiesis. HOTAIRM1 knockdown experiments on NB4 cells correlated with low expression of HOXA1 and HOXA4 cluster genes and block the expression of CD11b and CD18 during granulopoiesis. HOTAIRM1 expression is activated by all-trans retinoic acid, which induces the differentiation of myeloid progenitor cells to granulocytes and mature myeloid cells [53]. HOTAIRM1 transcript also interacts and form complexes with transcripts of other key chromatin structure modulating proteins such as CBX1, PRC1 and PRC2 [242]. HOTAIRM1 was overexpressed in NPM1-mutated AML. Furthermore amongst, 215, intermediate cytogenetics risk group AML patients, high HOTAIRM1 expression was associated with inferior overall survival (OR: 2.04; *P* = 0.001) and disease-free survival (OR:2.56; *P* < 0.001) and a higher cumulative incidence of relapse (OR:1.67; *P* = 0.046). Furthermore, high expression of HOTAIRM1 was associated with poor survival outcome in the subgroup of NPM1 mutation-positive AML patients [54]. HOXA-AS2: HOXA cluster antisense RNA 2 (HOXA-AS2) located between HOXA3 and HOXA4 genes in the HOXA cluster. Like HOTAIR and HOTAIRM1, HOXA-AS2 regulates differentiation of myeloblasts to mature granulocytes and myeloid cells [243]. Dong et al. proved the important role of HOXA-AS2 in chemoresistance of myeloblast and the lncRNA HOXA-AS2 could act as a therapeutic target for overcoming resistance to chemotherapy in AML [96].

DLEU1 and DLEU2 lncRNA mapped on the frequently deleted region of chromosome 3q14.3 region in lymphoma and leukemia. DLEU2 lncRNA act as pre miRNA for 15a and 16–1 and both are involved in the pathogenesis of CLL through NF-kβ activity [222, 223]. LincRNA-p21 in CLL is associated with p53 gene repression; thereby, it acts as tumor suppressor gene, and this finding was confirmed in 68 CLL patients, 62 MM patients when compared with 36 healthy controls. The correlation of p53 repression through LincRNA-p21 makes it eligible therapeutic and prognostic marker in CLL patients [216, 244].

BGL3 lncRNA regulates the oncogenic expression of BCR-ABL fusion gene through c-Myc mediated signaling. The expression of BGL3 gene was inversely regulated through miR-17, miR-93, miR-20a, miR-20b, miR-106a, and miR-106b in Philadelphia positive ALL and CML patients [57, 245].

Non-coding microRNAs (miRNAs) are posttranscriptional and posttranslational regulators of the target genes and proteins, respectively. The expression and modulation of target genes is disease and tissue-specific. In leukemia, miRNA expression signature depends upon the disease subtype, cytogenetic risk group, age and molecular lesions like fusion genes or various mutations in a gene like FLT3, cKIT, NPM1, BCR-ABL, MLL rearrangement. The most frequently deregulated miRNAs in CML include miR-10a, miR-17/92, miR-150, miR-203, and miR-328. Oncogenic role of miR-9 was suggested by Chen et al. in the subgroup of AML patients with mixed lineage leukemia (MLL)-rearrangement [246]. However, Emmrich et al. suggested tumor suppressor role and expression was down-regulated in pediatric AML with t (8;21) translocation [247]. A similar finding was observed in Fu et al. that miR-9-1 was down-regulated in t (8;21) AML patients [248]. Many recent studies have compiled the biological and clinical significance of miRNAs in acute and chronic leukemia [10, 240, 241].

Like lncRNA and miRNA, circular RNAs (circRNAs) express as housekeeping, and regulatory RNAs. The mode of action of circRNA may be autocrine or paracrine; therefore, these circRNAs have been detected in various body fluids. The circRNAs are stable in different body fluids like saliva, urine, blood, and CSF. The basal level of various circRNAs is crucial to explore for understanding their clinical significance. In leukemia, ultra-deep genomic data is available, which enabled to explore different ncRNA entities for their diagnostic and prognostic significance. Various types of circRNAs have been characterized based on their position in the gene, the intron origin circRNA and exonic circRNAs, intergenic circRNAs, and exon-intron circRNAs. Although, a various study has supported the notion of differential circRNA expression profile in leukemia but the validation data from experimental studies is limited.. The origin of circRNAs has been associated with fusion genes in leukemia [249]. Isolated studies have shown the role of following cirRNAs, f-circPR, f-circM9, hsa_circ_0075001, circ-ANAPC7, circ-100,290, circPAN3, circ_0009910, circ-HIPK2, circ-DLEU2, has_cir_0004277, circPVT1 in AML [59, 71, 250–252]. In CML, the direct association of circBA9.3 with BCR-ABL tyrosine kinase activity was observed in CML patients. The high expression of circBA9.3 was associated with cell proliferation and inverse relations with apoptosis. Furthermore, the high expression was associated with relapse and disease progression suggesting the possible role of circBA9.3 as a potential therapeutic marker in CML [72].

## Conclusions & future perspectives

The crucial role of ncRNAs in the gene regulatory networks and recent progress in the field of genomics and biotechnology has made them a favorable therapeutic targeting agent in cancer. lncRNAs and circRNAs act through various mechanisms as compared to miRNAs in cancer, and so targeting them can help in exploring more critical mechanisms involved in tumorigenesis. This review highlights the therapeutic potential of ncRNAs such as miRNAs, lncRNAs and circRNAs in leukemia and culminates the significance of these biomolecules as they improved the prognostic risk stratification in leukemia. The improvement in risk stratification has led to the generation of medical algorithms that can help in standardizing selection and treatment planning based on the molecular profile of the patient. These risk stratification schemes can be taken one step further by the inclusion of selected ncRNA expression profiles.

Additionally, by artificially modulating the expression of ncRNAs, the therapeutic sensitivity to conventional chemotherapy can be restored. In this regard, miRNAs have become the most extensively studied ncRNAs in leukemia because of their role as an oncogene and tumor suppressor in various cancers, including leukemia and their involvement in the regulation of post-transcriptional processes. The advanced genomic approaches, such as CRISPR-Cas9 technology is used to identify functionally relevant miRNA-mRNA target pairs that regulate leukemia (e.g., AML) cell line growth and will likely prove beneficial for preclinical models. Another approach is the use of miRNA mimics or modified miRNAs as RNA based drugs to target ncRNAs and mRNAs. Silencing of aberrant miRNAs can also be achieved by miRNA sponges and anti-miRNA oligonucleotides (AMOs). Finally, miRNA analysis through advanced next-generation sequencing will provide more details on the involvement of ncRNAs in the onset and progression of leukemia. For efficient miRNA-based therapy, improvised miRNA delivery vehicles with higher stability and less toxicity must be developed.

On the other hand, oncogenic lncRNAs can be targeted using siRNAs by packaging them in nanoparticle vectors for efficient targeting. In addition, high affinity or stability of antisense oligonucleotides can be achieved by synthetically modifying themto reduce the oncogenic lncRNAs by alternative splicing, modulation of RNA and protein interactions or by degrading them. Further, lentiviral vectors can be used as an efficient method for the transportation of RNA products into tissues as they aid in stable transfection by efficiently inserting the siRNA sequence into target cells.

Regarding the many roles of ncRNAs in cancer, there are still many challenges that must be resolved in order to improve the potential of ncRNAs as a potential therapeutic target in cancer. As the complex microenvironment of the cell makes the delivery of ncRNAs very challenging and difficult, the efficient delivery system with minimal toxicity is vital. It is suggested that the drug delivery can be improved by using two or more different carriers for targeting ncRNAs, for example combining nano designs with organ-specific response receptor. Moreover, in order to increase their bioavailability, different ways must be discovered to reduce RNA degradation. Although the field of ncRNAs is well studied, their role as a biomarker and as a therapeutic target in cancer is yet to be explored in detail. Many clinical trials are currently underway, and if some of the challenges mentioned above are addressed appropriately, then we would likely see ncRNAs emerging as a novel target for cancer therapy.

## Data Availability

Not applicable, please refer to the original reference.

## Abbreviations

ALL: Acute lymphoblastic leukemia
AML: Acute myeloid leukemia
ATRA: All-trans retinoid acid
BCLAF1: BCL2-associated transcription factor 1
BGL3: Beta Globin Locus 3
CD47: Cluster of differentiation 47
ceRNA: Endogenous RNA
circRNA: Circular RNA
CLL: Chronic lymphoblastic leukemia
CML: Chronic myeloid leukemia
CRC: Colorectal cancer
DCs: Dendritic cells
DLEU1: Deleted in lymphocytic leukemia 1
DLEU2: Deleted in lymphocytic leukemia 2
eRNAs: enhancer RNAs
GVHD: Graft-versus-host disease
HOTAIRM1: HOXA transcript antisense RNAs, myeloid-specific 1
HOXA-AS2: HOXA cluster antisense RNA2
lncRNA: long non-coding RNA
MDR: Multidrug resistance
miRNA: microRNA
MSC: Mesenchymal stromal cells
ncRNA: non-coding RNA
NEAT1: Nuclear enriched abundant transcript 1
PD-L1: Programmed death-ligand 1
PI3K: Phosphoinositide-3 kinase
PRC2: Poly-comb repressive complex-2
pre-miRNA: precursor miRNAs
Pri-miRNA: Primary miRNA
PVT1: Plasmacytoma variant translocation 1
siRNAs: small interfering RNAs
TME: Tumor microenvironment
