# Correction to: Role of non-coding RNA networks in leukemia progression, metastasis and drug resistance

**DOI:** 10.1186/s12943-020-01303-5

**Published:** 2020-12-29

**Authors:** Ajaz A. Bhat, Salma N. Younes, Syed Shadab Raza, Lubna Zarif, Sabah Nisar, Ikhlak Ahmed, Rashid Mir, Sachin Kumar, Surender K. Sharawat, Sheema Hashem, Imadeldin Elfaki, Michal Kulinski, Shilpa Kuttikrishnan, Kirti S. Prabhu, Abdul Q. Khan, Santosh K. Yadav, Wael El-Rifai, Mohammad A. Zargar, Hatem Zayed, Mohammad Haris, Shahab Uddin

**Affiliations:** 1 Functional and Molecular Imaging Laboratory, Cancer Research Department, Sidra Medicine, P.O. Box 26999, Doha, Qatar; 2 Department of Biomedical Science, College of Health Sciences, Qatar University, Doha, Qatar; 3 Translational Research Institute, Academic Health System, Hamad Medical Corporation, P.O. Box 3050, Doha, Qatar; 4 Laboratoryfor Stem Cell & Restorative Neurology, Era’s Lucknow Medical College and Hospital, Lucknow, Uttar Pradesh India; 5 Department of Medical Lab Technology, Faculty of Applied Medical Sciences, University of Tabuk, Tabuk, Saudi Arabia; 6 Department of Medical Oncology, Dr. B. R. Ambedkar Institute Rotary Cancer Hospital, All India Institute of Medical Sciences, New Delhi, India; 7 Department of Biochemistry, Faculty of Science, University of Tabuk, Tabuk, Saudi Arabia; 8 Department of Surgery, Sylvester Comprehensive Cancer Center, Miller School of Medicine, University of Miami, Miami, Florida USA; 9 Department of Biotechnology, Central University of Kashmir, Ganderbal, Jammu and Kashmir India; 10 Laboratory Animal Research Center, Qatar University, Doha, Qatar

**Correction to: Mol Cancer 19, 57 (2020)**

**https://doi.org/10.1186/s12943-020-01175-9**

Following the publication of the original article [1], authors have noticed that one of the authors, Syed Shadab Raza, was missing in the Authors’ Contributions section. Please see below updated section.

**Authors’ contributions**

Conceptualization, AAB, SYN, MH and SU; writing—original draft preparation, AAB, SN, IA, RM, SKS, LZ, IE, SK, KSP, AQ, SK and SU; writing—review and editing, AAB, SN, MH, MK, WER, HZ, MAZ and SU; Revision of manuscript, AAB, SSR, SN, IA, RM, SKS, LZ, IE, SK, KSP, AQ, SK, SKY and SU; supervision, AAB, SU, MH, SN, MK, WER and SU. AAB, SSR and LZ —Preparation of Illustrations. All authors have read and approved the final version of the manuscript.
